# The Treatment with Complementary and Alternative Traditional Chinese Medicine for Menstrual Disorders with Polycystic Ovary Syndrome

**DOI:** 10.1155/2021/6678398

**Published:** 2021-05-17

**Authors:** Yuehui Zhang, Xiaozhu Guo, Shuting Ma, Haoyue Ma, Hang Li, Yi Wang, Zhen Qin, Xiaoke Wu, Yaguang Han, Yanhua Han

**Affiliations:** ^1^Department of Obstetrics and Gynecology, The First Affiliated Hospital of Heilongjiang University of Chinese Medicine, Harbin, China; ^2^Heilongjiang University of Chinese Medicine, Harbin, China

## Abstract

Polycystic ovary syndrome (PCOS) is a frequent gynecological female endocrinopathy, characterized by chronic anovulation, hyperandrogenism, and insulin resistance (IR). Menstrual disorders are one of the main clinical manifestations of PCOS. Other symptoms include hirsutism and/acne. At present, the treatment of PCOS with irregular menstruation is mainly based on oral contraceptives, but there are some side effects and adverse reactions. In recent years, more and more attention has been paid to the complementary and alternative medicine (CAM), which has been widely used in clinical practice. Modern Western medicine is called “conventional medicine” or “orthodox medicine,” and the complementary and alternative medicine is called “unconventional medicine” or “unorthodox medicine.” CAM includes traditional medicine and folk therapy around the world. Around 65–80% of world health management business is classified into traditional medicine by the World Health Organization, which is used as alternative medicine in Western countries. In our country, Chinese medicine, acupuncture, and other therapies are commonly used due to their significant efficacy and higher safety. Therefore, this review aims to summarize and evaluate the mechanisms and the effect of current complementary replacement therapy in the treatment of menstrual disorders caused by PCOS, so as to provide guidance for the following basic and clinical research.

## 1. Introduction

PCOS is a common gynecologic endocrine disease, characterized by high androgen, persistent anovulation, and polycystic ovary changes. The prevalence of PCOS in women of childbearing age ranges from 6% to 21% [1],; however, the cause of this disease is still unclear [2, 3]. As one of the main clinical symptoms of PCOS, menstrual disorders mainly include premenstrual period, post-menstrual period, menorrhagia, less menstrual flow, and other types, which afflict women of all ages. The study by Rajiwade et al. [4] found that adolescent girls are easily affected by anxiety and other factors due to the rapid transformation of their physical development, which leads to endocrine and metabolic disorders, and finally menstrual abnormalities. In addition, an article written by Rostami et al. [5] also summarized and proposed that women have irregular menstruation due to the dysfunction of the ovario-thyroid axis. The menstrual disorder caused by PCOS are mainly caused by the complex endocrine characteristics of PCOS, high blood androgen level, elevated luteinizing hormone/follicle-stimulating hormone (LH/FSH) ratio, and excessive insulin, which will affect ovulation of the ovary and lead to menstrual disorders. At present, the treatment for menstrual disorders caused by PCOS is mainly based on drug therapies. The first-line treatment drugs are oral contraceptives; but, these may have adverse effects on glucose tolerance, fertility, and so on [6–8]. At the same time, experimental have data confirmed that long-term usage of contraceptives has a close relationship with hypertension, venous thrombosis, breast cancer, and other cancers [9–12]. Therefore, it is particularly important to select a complementary alternative therapy, which has high safety and more selectivity, and few reports of adverse reactions [13].

Nowadays, CAM is getting more and more attention from all over the world, which also plays a great role in the prevention and treatment of diseases. The rapid development of CAM techniques in TCM diagnosis and treatment has provided more options for complementary and alternative treatments. When facing illness, not only the Asian people will seek help from CAM including TCM but also a large portion of Western people will pick it as one of their choices [14]. The paper summarized and evaluated the mechanisms and effects of current TCM complementary and alternative therapies, such as monomers, compounds, acupuncture, auricular acupoints, massage, Qigong, Tai Chi, cognitive behavioral therapy, and healthy lifestyle, in the treatment of menstrual disorders caused by PCOS, so as to provide guidance for subsequent basic and clinical research.

## 2. The Treatment with Traditional Chinese Medicine in PCOS with Menstrual Disorders

TCM, as a major part of CAM, plays an irreplaceable role in the prevention and treatment of diseases whose existence is irreplaceable and unrepeatable. Recently, with the culture of TCM spreading all over the world, it is being widely known and applied in practice. Moreover, it also exerts its unique advantages in the treatment of PCOS. According to relevant data, a survey of 493 Australian women diagnosed with PCOS found that more than 70% of these women chose to use CAM [15]. Generally speaking, TCM is divided into the monomer of Chinese medicine and compound Chinese medicine. For a long time, TCM has been used to treat gynecological diseases and its application is gradually increasing. The following paragraphs will describe and analyze the treatment of TCM in PCOS with menstrual disorders according to the monomer and compound of Chinese medicine.

### 2.1. The Monomer of Chinese Medicine

#### 2.1.1. Cryptotanshinone

Cryptotanshinone (CRY) is an effective monomer extracted from the stem root of Salvia miltiorrhiza [16]. TCM believes that *Salvia miltiorrhiza* has the function of a blood-activating, menstruation-regulating, stasis-dispelling analgesic, cooling the blood, eliminating the carbuncle, and acting as an anxiety-reliving tranquilizer. In modern medicine, Salvia miltiorrhiza, regarded as a kind of precious Chinese herbal medicine with the function of protecting heart and brain vessels, is widely used in treating cardiovascular diseases in Asian countries, including coronary heart disease, myocardial infarction, angina pectoris, and atherosclerosis because it is safe [17]. In addition, it can also improve the condition of acne, lupus erythematosus, and melanoma [18–20]. According to the recent studies, specialists have found that Salvia miltiorrhiza also has anticancer activity, which can be used to treat cancers like lung cancer [21–24]. As one of the most important extractions, CRY plays a significant role in the prevention and treatment of diseases like PCOS, which cannot be underestimated. With regard to treating PCOS, CRY can improve the statement of hyperandrogen in PCOS patients through a variety of methods. Clinical trials have confirmed that CRY has a curative effect on PCOS patients. In Shen et al.'s study [25], 100 patients were randomly assigned to tanshinone or placebo group, with 50 patients in each group. Tanshinone or placebo capsules were given orally for 12 weeks, and the main outcome parameters, such as plasma testosterone (T), human chorionic gonadotropin-induced androgen response, IR, reproductive hormone, fasting blood lipid, oral glucose tolerance test, quality of life, and side effects were recorded and compared. The results showed that all indicators were significantly improved after taking CRY compared with the placebo group.

In the animal experiment conducted by Yu et al. [26], PCOS model rats were induced by subcutaneous injection of dehydroepiandrosterone (DHEA), observing the weight and ovarian morphology of rats after the CRY interventions and using the RIA method to detect serum biochemical indexes. The results showed that CRY could significantly recover the estrus of PCOS rats, and the serum biochemical indexes were also improved. It was proved that CRY could rebalance the reproductive and metabolic disorders of PCOS rats by regulating the expression of CYP 17 and AR, so as to restore the menstrual cycle. According to an experiment conducted by Yang et al. [27], PCOS rats model was established by injecting human chorionic gonadotropin and insulin daily for 22 days. Compared with the control group, the weight of PCOS rats, ovarian weight, and LH were significantly increased. It was proved that CRY could protect against PCOS-induced damage of ovarian tissue, possibly through a regulatory pathway involving HMGB1, TLR4, and NF-*κ*B. At the same time, it can also recover the regular menstruation by protecting the functions of the ovary from being hampered. In addition to the above mechanism, the experiment conducted by Ye et al. [28], granulosa cells (PGCs) were isolated from porcine ovary to culture and conduct experiments. The results showed that CRY could regulate androgen synthesis of porcine granulosa cells through ERK/c-fos/CYP 17 pathway. All the basic experiments mentioned above proved that CRY has a therapeutic effect on the hyperandrogenesis in PCOS patients.

#### 2.1.2. Berberine

Berberine (BBR) is an alkaloid separated from Chinese medicinal Coptis chinensis. It is the main active component of Coptis chinensis. TCM holds the thought that Coptis chinensis has functions of reducing body heat, drying dampness, purging fire, and detoxification. Owing to the antibacterial and other functions of BBR, according to the modern pharmacological research, it is widely used in TCM for anti-infection, hypercholesterolemia, type 2 diabetes, and other diseases [29]. At the same time, BBR also plays a significant role in improving metabolism. Besides, BBR can also be used to treat IR in patients with PCOS. Studies have shown that there appeared no such serious adverse reactions but gastrointestinal reactions [30] compared with the common treatment of Western medicine. According to the related literature, BBR treating women with PCOS menstrual disorder is concerned with the improvement of IR [31–35]. Experiments showed that BBR plays the same role as metformin in improving IR [31, 32], which means BBR can improve obesity by improving insulin level in vivo, thereby regulating menstruation [29]. In the experiment of Orio et al. [33], a total of 100 people, that is, 50 obese PCOS patients were selected as the experimental group, and 50 menopausal healthy women who were matched with age and weight were selected as the control group. BBR was given to the experimental group for six months. The results showed that BBR could significantly increase the menstrual frequency; at the same time, the increasing of the SHBG and total T in PCOS patients also proved that BBR could improve the metabolism and hormone level of PCOS patients [34]. In addition, BBR is superior to metformin in improving IR and safety in PCOS patients. A randomized controlled trial was conducted by An et al. [35] with 150 PCOS infertile women. They were randomly divided into the BBR group, metformin group, and placebo group, with 50 women in each group. This experiment lasted for 12 weeks with results showing that the recovery of ovarian function was reflected by in vitro pregnancy, and indirectly reflected the improvement of irregular menstruation. Apart from that, improvement of BMI, TC, LDL, and other indicators in BBR group was better than that in the metformin group and placebo group. Moreover, the adverse events of gastrointestinal tract in BBR group were less than the metformin group, and the survival rate of in vitro pregnancy was higher than the other two groups. It was concluded that BBR was safer for premenopausal women at the same time giving the least stimulation to ovary. BBR could improve the metabolism of ovarian stimulation and the response to ovarian stimulation. Comparatively speaking, BBR can significantly improve PCOS menstrual disorder [34]; it is also effective in improving IR in PCOS patients and regulating menstrual cycle by improving metabolism.

Zhang et al. [36] showed that BBR could improve glucose transporter 4 (GLUT4) and reduce IR in PCOS rats through dual regulation of PI3K/Akt and MAPK. By measuring the protein levels of GLUT4, PI3K/Akt, and MAPK dual pathway, it can help to restore the HOMA-IR and ISI values to the normal level, and enhance the expression of GLUT4, so as to restore the normal ovarian function and menstrual regularity.

We find that the experiment conducted by Zhang [37] studied the pharmacologic effects of Diane-35, probiotics, and BBR on PCOS patients from the perspective of intestinal microflora. The authors had consulted other experiments showing that BBR could improve IR of PCOS patients during the experiment. In their experiment, they compared three treating methods including Diane-35 (estrogen and progesterone), probiotics, and BBR. They found that Diane-35 and probiotics could improve the reproductive and metabolic functions in the PCOS rats along with restoring the diversity of the gut microbiota leading to the improvement of the reproductive function in PCOS-like rats. As for the BBR administration to PCOS rats, the composition and diversity of intestinal microflora were significantly reduced, showing no improvement in terms of metabolism or reproduction Phenotypic in PCOS, which was contrary to the previous findings.

#### 2.1.3. Cinnamon

As one of the Chinese herbal medicines, cinnamon has the functions of tonifying fire, helping Yang, dispersing cold and relieving pain, activating blood, and unblocking meridians. Moreover, as a common edible spice, it is considered to have anti-PCOS and anti-diabetes characteristics [38]. Cinnamon can prevent IR caused by high fructose diet, and early injection can prevent the development of IR [39]. Many clinical trials have also shown that cinnamon can improve metabolism by improving IR. For example, in the placebo-controlled, double-blind, randomized trial conducted by Kort and Lobo [40], 45 women were randomly selected to receive cinnamon replacement therapy. Among them, 26 women completed the 3-month study and the remaining 17 women completed the 6-month study. The results showed that the menstrual cycle of the patients taking cinnamomum cassia was more frequent than those taking placebo, and the improvement of the menstrual cycle was more obvious in women who took for 6 months, suggesting that cinnamon supplement can improve menstrual cycle. Hajimonfarednejad et al. [41] conducted a randomized, double-blind, placebo-controlled clinical trial on cinnamon powder in 66 patients with PCOS. It was also proved that cinnamomum cassia can significantly reduce fasting insulin and IR in PCOS patients. In the experiment [42] of Wang et al., 15 patients with polycystic bursa were randomly divided into oral cinnamon and placebo group for 8 weeks. After treatment, compared with the baseline insulin sensitivity index of fasting and 2-hour oral glucose tolerance test, IR in cinnamon group was significantly reduced, while that in placebo group was not. It was found that cinnamomum cassia could enhance the effect of insulin by increasing the activity of phosphatidylinositol 3-kinase in insulin signaling pathway.

In the experiment of Qin et al. [38], the rats were divided into oral saline group and cinnamomum cassia group, comparing the glucose infusion rate after insulin 3 mU/kg was injected. With the increase in insulin injection volume proportion, the glucose infusion rate of cinnamomum cassia group was significantly raised. Compared with other groups, such as IR -*β* and IRS-1 tyrosine phosphorylation level and IRS-1/PI-3 kinase combined with oral cinnamon group, the results showed that cinnamon extraction can improve the effect of insulin by increasing glucose uptake in vivo. Furthermore, it can be concluded that cinnamomum cassia can improve IR and regulate the metabolic level of the body, so as to improve the menstrual disorder of PCOS patients. Referring to the relevant literature, in the basic experiment [43] conducted by Dou et al., 60 female mice were randomly divided into three groups for 20 days, with 20 mice in each group. Blank group: injected sesame oil and methylcellulose; model group: injected DHEA and methylcellulose to establish PCOS mouse model; experimental group: injected equal amount of DHEA and cinnamon powder mixed with methylcellulose. The three groups were given insulin intraperitoneal injection 20 days later for glucose tolerance test. Body weight was observed at any time during the experiment, and reproductive and metabolic characteristics were evaluated. After testing the levels of T and serum insulin, the results showed that cinnamomum cassia could restore the cell cycle and ovarian morphology of PCOS mice induced by DHEA. In addition, cinnamomum cassia also inhibited the expression of IGF-1 and IGFBP-1 induced by DHEA. It is suggested that cinnamomum cassia can be used as a supplementary treatment to improve IR, restore ovarian function, and improve menstrual disorder in PCOS mice.

However, the placebo-controlled, double-blind, randomized trial conducted by Kort and Lobo [40] reported four nonserious adverse events, including headache, heartburn, menstrual cramps, nausea, and diarrhea. In this paper, [44] speculated that the occurrence of adverse reactions may be related to variable dose.

### 2.2. Compound Chinese Medicine

#### 2.2.1. Cangfu Daotan Decoction

Cangfu Daotan decoction [45] is a TCM prescription widely used in patients with PCOS of phlegm and dampness type. Its original prescription is Atractylodis Rhizoma, Cyperi Rhizoma, Aurantii Fructus Immaturus, Citri Unshius Pericarpium, Poria Sclerotium, Arisaematis Rhizoma Preparata cum Bovis Fel, and Glycyrrhizae Radix et Rhizoma. It has the effect of removing the phlegm and dampness. There are many randomized controlled clinical trials for the treatment of PCOS patients with Cangfu Daotan decoction. Yang's randomized controlled clinical trial [46] showed that Cangfu Daotan decoction had a significant effect on late menstruation caused by hyperinsulinemia of kidney deficiency and phlegm dampness type; 58 patients who met the diagnostic criteria were randomly divided into two groups, 29 cases in each group. The treatment group was treated with Cangfu Daotan decoction. The prescription consists of Atractylodes lancea, Rhizoma Cyperi, Pericarpium Citri Reticulatae, Pinellia ternata, Poria cocos, dannanxing, Zhishi, jinneijin, silkworm excrement, raw hawthorn, Cuscuta chinensis, epimedium, Cistanche, antler tablets, Morinda officinalis, and the control group was treated with metformin. The total effective rate was 82.7% in the treatment group and 65.5% in the control group. In clinical application, Cangfu Daotan decoction is usually used in combination with conventional Western medicine for the treatment of PCOS. Huang's randomized controlled clinical trial [47] showed that Cangfu Daotan decoction combined with clomiphene has a good long-term effect in treating PCOS amenorrhea of phlegm dampness type. There were 68 cases of PCOS, 35 cases in the treatment group and 33 cases in the control group. The control group was given clomiphene, and the treatment group was treated with Cangfu Daotan decoction on the basis of the control group. The prescription is Atractylodes Rhizoma, Rhizoma Cyperi, Pinellia ternata, Poria cocos, tangerine peel, dannanxing, Fructus aurantii, ginger, and liquorice. The course of treatment in both groups was 6 menstrual cycles. The levels of LH, prolactin (PRL), and T in the two groups were significantly decreased, and the short-term effect of the treatment group was similar to that of the Western medicine control group. However, 3 months after the drug withdrawal, the hormone level of the control group returned to the level before treatment, the treatment group remained at the level after treatment, and the long-term total effective rate of the treatment group was significantly higher than that of the control group. Hua et al.'s randomized controlled clinical trials [48] showed that Cangfu Daotan decoction (Rhizoma Cyperi, Atractylodes lancea, Rhizoma Pinelliae, Acorus tatarinowii, Gleditsia sinensis, Pericarpium Citri Reticulatae, Poria cocos, Xianlingpi, Yam, Astragalus membranaceus, Angelica, and Salvia miltiorrhiza) combined with Dian-35 and metformin can reduce the menstrual volume, infertility, obesity, hairiness, and other TCM Syndromes of PCOS patients, improve the levels of sex hormone and blood lipid, and reduce insulin resistance index (HOMA-IR). Du's clinical trial [49] showed that Cangfu Daotan decoction (Atractylodes macrocephala, Rhizoma Cyperi, Poria cocos, Fructus aurantii, Pericarpium Citri Reticulatae, dannanxing, Rhizoma Pinelliae, Atractylodis Macrocephalae, coix seed, Chinese yam, prepared radix rehmanniae, dodder seed, antler gum, Angelica sinensis, Ligusticum chuanxiong, and Glycyrrhiza uralensis) combined with Diane-35 can improve TCM syndrome, basic sex hormone, menstrual cycle, menstrual volume, and polycystic ovary of PCOS.

Yi et al.'s experiment [50] showed that Cangfu Daotan decoction decreased the serum levels of TCHO, TG, LDL-c, LH, T, IL-1*β*, IL-6, and TNF-*α* and increased the levels of HDL-c, follicle-stimulating hormone (FSH), and estradiol (E2) in PCOS rats model. The mechanism of Cangfu Daotan Decoction in the treatment of PCOS may be related to its regulation of lipid metabolism, sex hormone secretion, inflammatory reaction, and induction of the expression of OATP2B1 and OATP3A1 in ovarian and uterine tissues. Xu et al.'s research [51] showed that 111 active components were extracted from 1433 components of Cangfu Daotan decoction, involving 118 protein targets. 736 genes were found to be closely related to PCOS, and 44 of them overlapped with Cangfu Daotan decoction. Pathway enrichment analysis identified the AGE-RAGE signaling pathway in diabetic complications, endocrine resistance, the IL-17 signaling pathway, the PRL signaling pathway, and the HIF-1 signaling pathway. In addition, PI3K-Akt, IR, Toll-like receptor, MAPK, and AGE-RAGE were related to the treatment of PCOS.

#### 2.2.2. Others

Li et al.'s experiments [52] have shown that the long-term efficacy of TCM in the treatment of PCOS menstrual related symptoms is more stable and lasting than that of Western medicine. In the randomized controlled clinical trial of Li et al., 66 patients with PCOS were randomly divided into TCM group and Western medicine groups, with 33 cases in each group. TCM group was treated with modified Zigui Decoction (Prescription: Cornus officinalis, Fructus Ligustri Lucidi, Herba Ecliptae, dodder, radix rehmanniae, Radix Paeoniae Alba, amethyst, and Xianlingpi) and the Western medicine group was treated with Diane-35. The normal rate of menstrual cycle in the TCM group was 57.58% after one menstrual cycle; 63.64% in the Western medicine group; 45.45% in the TCM group after six menstrual cycles; 21.21% in the Western medicine group; and the TCM group was better than that of the Western medicine group. After one menstrual cycle, the volume of ovaries in both groups decreased significantly; the number of follicles was significantly reduced, and the ratios of LH, T, and LH/FSH were lower than that before treatment. After 6 menstrual cycles, the volume of bilateral ovaries in the TCM group was still significantly reduced compared with before treatment, while the Western medicine group returned to the state before treatment; The number of follicles in the TCM group was still significantly reduced, and the Western medicine group was restored to the state before treatment; the hormone level in the TCM group still decreased significantly, and the Western medicine group returned to the level before treatment. The clinical trials conducted by Lai et al. [53] showed that the TCM with the prescription of Paeonia lactifloraPall, Bupleurum chinenseDC, Citrus reticulatataBlanco, Ligusticum chuanxiongHort, Angelica sinensis (Oliv.) Diels, Glycyrrhiza uralensisFisch, Lycium barbarumL, Cinnamomum cassiaPresl, Carthamus tinctoriusL, Prunus persica (L.) Batsch, Cuscuta chinensisLam, Cyperus rotundusL, Leonurus japonicusHoutt, Citrus aurantiumL can improve the menstrual regularity of PCOS patients, including from amenorrhea to hypomenorrhea or menorrhagia, from hypomenorrhea to menorrhagia, or pregnancy. In addition, the modified ferriman Gallwey score was significantly lower than that before treatment, and hirsutism was improved. The data of liver and kidney function and adverse events were basically normal. This prescription has the function of soothing liver and replenishing qi, tonifying the kidney yang, nourishing blood, and activating blood circulation. The randomized controlled clinical trial of Liu and Mao [54] showed that Danzhi Xiaoyao Pill (Radix Bupleuri, Angelica sinensis, Radix Paeoniae Alba, Atractylodes Macrocephalae, Poria cocos, Glycyrrhiza uralensis, cortex moutan, and Gardenia jasminoides) can improve the ovulation rate of PCOS patients with IR, and has better curative effect in improving symptoms such as irritability, chest tightness, hypochondria, premenstrual chest pain, bitter mouth, dry mouth, less menstruation, menstrual color, abnormal menstruation frequency, pulse, tongue, etc., and has obvious advantages over Western medicine alone. Tao et al.'s randomized controlled clinical trial [55] showed that modified Longdan Xiegan decoction (Gentian grass, Scutellaria baicalensis, Gardenia jasminoides, Alisma orientalis, plantain seed, Angelica sinensis, Shengdi, bupleurum, Cortex Moutan, and Prunella vulgaris) could significantly improve the symptoms of PCOS patients of stagnant fire in Gan channel type, menstrual disorder, acne, and basal body temperature, and the adverse reactions were lower than those in the Western medicine group. Hou et al.'s randomized controlled clinical trial [56] showed that Tiangui decoction (Anemarrhena asphodeloides, Ophiopogon japonicus, Polygonatum sibiricum, Angelica sinensis, Psoralea corylifolia, Rhizoma Polygoni cuspidatum, Verbena officinalis, Xianlingpi, Shengdi, and Taoren) can reduce the high insulin concentration of PCOS patients, induce ovulation, and resume menstrual cycle. The efficacy of Tiangui Capsule, a kind of TCM compound, in the treatment of PCOS was evaluated by a randomized controlled clinical trial by Kuek and compared with metformin and Diane-35 [57]. The effect of Tiangui Capsule on hyperandrogenemia was not as good as Diane-35, but better than metformin. Tiangui capsule is not as effective as metformin in the treatment of hyperinsulinemia, but better than Diane-35. It was concluded that Tiangui capsule can treat PCOS by regulating ovarian function and reducing the insulin level.

Zhao et al.'s experiment [58] showed that the therapeutic effect of Heqi San (*Curculigo orchioides* Gaertn, *Schisandra chinensis* (Turcz.) Baill, *Cynanchum otophyllum* C. K. Schneid, *Citrus medica* L. var. sarcodactylis Swingle, *Crataegus pinnatifida* Bunge, *Rhus chinensis* Mill, *Clinopodium megalanthum* (Diels) C. Y. Wu & Hsuan ex H. W. Li, *Cuscuta chinensis* Lam, *Poncirus trifoliata* (L.) Raf, *Hordeum vulgare* L, *Polygala tenuifolia* Willd, and *Epimedium davidii* Franch) on the rats model of PCOS includes altering serum hormone levels, healing ovary morphological lesions, and improving IR, which is mediated through the PI3K/Akt pathway. Azeemuddin's study [59] evaluated “DXB-2030,” a polyherbal combination of Trigonella foenum-graecum, Aloe vera, Sphaeranthus indicus, Nardostachys jatamansi, and Symplocos racemosa extracts in an experimental model of testosterone propionate (TP), which induced PCOS in female rats. Results showed that “DXB-2030” reversed the TP-induced changes by increasing the GLUT4 expression and decreasing the body weight, T levels, AUC of glucose in Oral Glucose Tolerance Test (OGTT), and the cystic follicles of the ovaries. The effect of “DXB-2030” on the reproductive system of women with PCOS may include reversion of estrus cyclicity, reduction in ovary volume and size of the cyst, antiandrogenic effect, decreased T levels, and restoration of the histology of ovarian tissue. Bushen Huatan decoction [60] can improve the serum metabolites in patients with PCOS, and has a certain therapeutic effect on PCOS by reducing inflammatory reaction and oxidative stress. In addition, Wang et al.' s experiment [61] has shown that Siwu decoction may has the effect of promoting follicular development and establishing normal menstrual cycle in patients with PCOS.

The current problem is that the sample size of randomized controlled trials is too small. In addition, most randomized controlled trials use the method of observing the treatment effect for three or six months, which means that the observation time needs to be extended. Lack of placebo control may affect the analysis of drug efficacy. The lack of blinding may affect the predicted treatment outcomes, especially subjective outcomes, such as self-reported quality of life, psychology, and compliance. Therefore, randomized controlled trials (double-blind placebo-controlled) are needed to verify the efficacy of the drug.

For summarizing the randomized clinical trials, please refer to Table 1.

## 3. Acupuncture Treatment for Menstrual Disorder in PCOS

Acupuncture, as the major part of TCM, the history of can be traced back to more than 3000 years [62,63], is based on the basic theory of TCM, selecting acupoints according to the syndrome differentiation and experience, thus achieving the purpose of treatment of diseases through a variety of manipulation. In recent years, due to the limited efficacy and common side effects of Western medicine in the process of treating PCOS patients [64], acupuncture plays an important role in the treatment of menstrual disorders caused by PCOS [65]. Therefore, acupuncture has been used as an alternative treatment for PCOS, which has gradually become a researching hot spot recently and received extensive attention [29]. As a kind of TCM treatment, acupuncture is divided into acupuncture, moxibustion, acupoint catgut embedding, and other categories. The following paragraphs will be discussing the acupuncture treatment for menstrual disorder in PCOS patients according to the different categories.

### 3.1. Acupuncture Treatment for PCOS Menstrual Disorder

#### 3.1.1. Clinical Application of Acupuncture for Menstrual Disorder of PCOS

As one of the complementary and alternative therapies, acupuncture is getting more and more attention in the treatment of PCOS patients with menstrual disorders [66], and there have been a number of reports on the efficacy of acupuncture in treating PCOS. However, there has been no study on the method and quality of standard reports on the effectiveness of acupuncture and moxibustion on PCOS [67–70]. In Zheng et al. ‘s randomized controlled clinical trial [71], 86 patients were randomly divided into abdominal acupuncture group and metformin group, with 43 patients in each group. The acupuncture group received 30 minutes of treatment twice a week.

**Figure 1:**
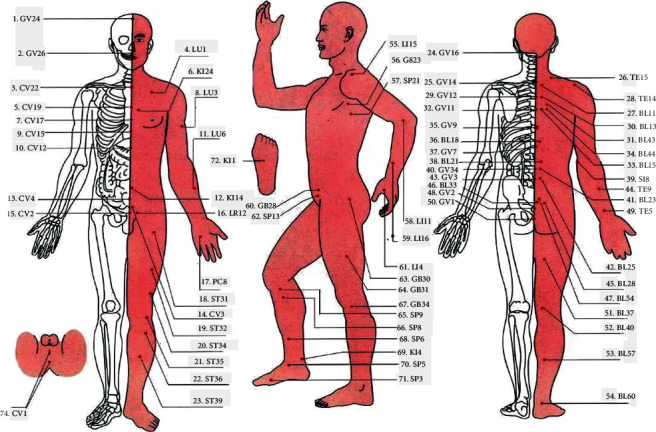
[81]. Human acupoint map GV24 Shengting, GV26 Shuigou, CV22 Tiantu, CV1 Zigong, CV17 Tanzhong, CV15 Jiuwei, CV12 Zhongwan, CV4 Guanyuan, CV2 Qvku, CV1 Huiyin, LU1 Zhongfu, KI24 Lingxu, LU3 Tianfu, LU6 Kongzui, PC8 Laogong, ST31 Biguan, CV3 Dahe, ST32 Futu, ST34 Liangqiu, ST35 Dubi, ST36 Zusanli, ST39 Xiajuxu, GB28 Weidao, SP13 Fushe, KI14 Siman, LR12 Qichong, LI15 Jianyu, GB23 Zhejin, SP21 Dabao, LI11 Quchi, LI16 Pianli, LI4 Hegu, GB30 Huantiao, GB31 Fengshi, GB34 Yanglingquan, SP9 Yinlingquan, SP8 Diji, SP6 Sanyinjiao, KI4 Dazhong, SP5 Shangqiu, SP3 Taibai, GV16 Fengfu, GV14 Dazhui, GV12 Shengzhu, GV11 Shendao, GV9 Zhiyang, BL18 Ganshu, GV7 Zhongshu, BL21 Weishu, GV4 Mingmen, GV3Yangguan, BL33Zhongliao, GV2 Yaoshu, GV1Changqiang, TE15 Tianliao, TE14 Jianliao, BL11 Dashu, BL13 Feishu, BL43Gaohuang, BL44 Shengtang, BL15 Xinshu, SI8 Xiaohai, TE9 Sidu, BL23 Shenshu, TE5 Waiguan, BL25 Dachangshu, BL28 Pangguangshu, BL54 Zhibian, BL37 Yinmen, BL40 Weizhong, BL57 Chengshan, and BL60 Kunlun.

After 6-month-treatment, in terms of reducing BMI and increasing menstrual frequency, the abdominal acupuncture group appeared better than the metformin group. Both abdominal acupuncture and metformin can improve the endocrine and metabolic functions of obese PCOS patients. Nevertheless, the advantages of the abdominal acupuncture are more outstanding apparently, which reflected in less side effects, reducing BMI significantly by reducing visceral adipose tissue [72]. In Li et al.'s double-blind, placebo-controlled, multicenter randomized controlled study [73] subsequently, 342 patients with PCOS and IR were randomly divided into three groups: true acupuncture + metformin placebo, sham acupuncture + metformin, and sham acupuncture + metformin placebo, with 114 patients in each group. Results of the oral glucose tolerance test and insulin release test collected 3 months after treatment showed that acupuncture could improve the sensitivity of insulin in PCOS patients. The IR of PCOS is caused by hyperinsulinemia and deficiency of insulin signaling pathway; therefore, high concentration of the insulin reduces the circulating level of SHBG and increases the level of free T, resulting in menstrual disorder [73,74]. Consequently, it is essential to reduce insulin levels. These two clinical trials have proved that acupuncture can improve the insulin sensitivity of PCOS patients, thereby improving hyperinsulinemia and relieving the symptoms of menstrual disorders. It has been found that acupuncture and moxibustion could regulate the reproductive and endocrine function of PCOS patients by affecting various signal pathways and targets of hypothalamus pituitary gonadal axis [75]. In Yu et al.'s randomized, drug-controlled, parallel grouping trial [76], they compared the efficacy and safety of Dong's acupuncture therapy and oral contraceptives on sex hormones in patients with PCOS; 60 women aged 18–45 years with PCOS were randomly divided into the acupuncture group and control group, with 30 patients in each group. In the acupuncture group, Fuke, Huanchao, Tianhuang, renhuang, Guanyuan, and uterus (EX-CA1) were selected, and the participants received acupuncture twice a week. After 12 weeks, compared with the control group, the LH/FSH ratio, LH, and T value in the acupuncture group were significantly reduced, indicating that acupuncture can effectively reduce the LH/FSH ratio, thereby regulating menstrual frequency and treating menstrual disorders caused by PCOS. Other studies have shown that low-frequency electroacupuncture can induce ovulation [77], thus making menstrual frequency return to normal. In addition, repeated electroacupuncture treatment can also reduce the ratio of LH/FSH and improve menstrual frequency [77]. The study found that women with PCOS had higher sympathetic nervous system activity [78]. In a prospective randomized controlled trial conducted by Jedel et al. [79], 84 women were selected and divided into the intervention group and low-frequency electroacupuncture group, with 42 patients in each group. Each participant experienced a 12-week-observation period. The results showed that serum T in the low frequency EA group was significantly lower than that in the intervention group, and menstrual frequency continued to improve. Therefore, low-frequency electrical stimulation reduces high sympathetic activity [80], which may be helpful in the treatment of hyperandrogenemia and oligomenorrhea/amenorrhea. In the clinical trial, Julia Johansson et al. conducted a prospective randomized controlled clinical study; 32 female patients with PCOS were divided into manual group and low frequency electrical stimulation acupuncture group, with 16 patients in each group. The acupuncture points were ST1, CV3, CV6, ST29, SP6, SP9, LI4, GV20, ST2, ST25, ST29, LR3, and PC6 (Figure 1), the ovulation frequency in the acupuncture group was higher than that in the control group, 10–13 weeks later. Normal ovulation frequency makes regular changes in endometrium, which further improves the menstrual cycle of PCOS patients [82]. In recent years, there have been a number of meta-analyses on acupuncture treatment of PCOS menstrual disorders. Although the conclusions of each study are different, they all enrich the specific clinical application of acupuncture as CAM. Among them, a systematic review summarized and evaluated the effective data for the acupuncture treatment of PCOS menstrual disorders, focusing on the menstrual rate, and found that acupuncture was more likely to improve the menstrual rate under low levels of evidence compared with the group which did not receive acupuncture and metformin [83].

Acupuncture and moxibustion in the treatment of menstrual diseases caused by PCOS have been constantly making meaningful explorations, but there are also some controversial parts. For example, some adverse reactions may occur in the process of acupuncture in clinical trials, such as pain, redness, hematoma, and nausea after acupuncture [84,85]. In addition, some researchers have proposed that superficial acupuncture, compared with true acupuncture, cannot be regarded as placebo control, for these control methods are not inert [86,87]. Relevant experiments have shown that both true acupuncture and sham acupuncture can improve the LH/FSH ratio of patients with PCOS, thus affecting menstrual frequency [84]. Several systematic reviews on acupuncture and moxibustion for PCOS have not yet made a comprehensive meta-analysis on the changes of menstrual cycle so far. Therefore, more prospective, large-scale, and highly rigorous randomized controlled trials are needed to demonstrate this point in the future basic research and clinical trials.

#### 3.1.2. Mechanism of Acupuncture in the Treatment of PCOS Menstrual Disorders

Acupuncture is divided into two parts, including hand-acupuncture and electro-acupuncture. Hand-acupuncture refers to the method of using the filiform needle to penetrate the skin and stimulate a specific anatomical position of the body, and rapid rotation of the thumb and index finger to stimulate manually to achieve the effect of obtaining qi [88]. Electro-acupuncture refers to a treatment in which the needle is connected to a trace of low-frequency pulsed current by an electroacupuncture machine and the needle obtains qi when it penetrates into the acupoint of the human body [89]. Studies have shown that acupuncture can regulate endogenous regulatory systems, including endocrine, sympathetic nervous system, and neuroendocrine system [62,63], thereby improving women's endocrine disorders, menstrual frequency, and reducing the level of sex hormones [90–92]. The mechanism of acupuncture treatment for menstrual disorders caused by PCOS is considered from the following aspects.

The first point is that acupuncture can improve insulin sensitivity and relieve IR [93–98]. Hyperinsulinemia and IR are the most important endocrine characteristics of PCOS, which can lead to the increasing of androgen in the body and affect the ovulation resulting in menstrual disorders. A study showed [96] that acupuncture can effectively improve the insulin sensitivity of PCOS rats induced by dihydrotestosterone. In this study, ST27, ST28, ST29, SP6, SP9 were selected to acupuncture rats. Compared with the control group without acupuncture, the former was significantly higher than the latter. Johansson et al. [93] carried out low frequency electroacupuncture treatment on PCOS rats model induced by dihydrotestosterone for 5–6 weeks; the acupuncture sites were abdominal and hind limb muscles. Then, the body weight, groin fat, insulin sensitivity, and other related indicators of the rats were measured. It was concluded that repeated low-frequency electroacupuncture can improve the insulin sensitivity in skeletal muscle and adipose tissue of rats. Increased insulin sensitivity can alleviate IR in PCOS rats, thereby indirectly improving menstrual disorders caused by PCOS.

The second point is that acupuncture can affect the hypothalamus-pituitary-gonadal-axis (HPG), thus affecting the release of hypothalamic gonadotropin-releasing hormone (GnRH) and the secretion of pituitary gonadotropin [86,99].. Patients with PCOS may have abnormal HPG making the pituitary gland more sensitive to GnRH, which results in excessive LH secreted by the ovary, stimulating ovarian stroma and theca cells, thus producing excessive androgen, finally resulting in impaired follicular development and menstrual disorders. Maliqueoet et al. [89] performed acupuncture on DHT-induced PCOS rats at the body nodes corresponding to ovarian nerve insufflations in rectus abdominis and triceps surae. After 5–6 weeks of acupuncture, the LH, T, progesterone, and estrus cycle were evaluated, and it was concluded that acupuncture and moxibustion can improve the level of gonadotropin and regulate the estrus cycle. In one study [100], female rats that were exposed to DHT increased the number of GnRH-expressing cells and the expression of androgen receptor (AR) protein in the hypothalamus. In DHT-induced rats, high-intensity but low-frequency electrical stimulation for 5 days a week, lasting for 4–5 weeks, restored the normal levels of GnRH and AR expression. The regulation of HPG by acupuncture can improve the hormone level of PCOS patients and restore their normal menstrual cycle.

The third point is that low frequency electroacupuncture can also improve ovarian morphology, estrus cycle, and AR protein expression in PCOS rats modeled by regulating sympathetic nervous system activity [101, 102]. Furthermore, acupuncture has a significant effect on reducing body mass index of PCOS women [90], and it can also activate the physiological process similar to physical exercise to reduce obesity, so as to improve the physical quality and endocrine environment of PCOS patients and regulate the menstrual cycle [96].

### 3.2. The Application of Moxibustion in the Treatment of Menstrual Disorders in PCOS Patients

Moxibustion, as one of the traditional medical therapies in China, prevailed in the spring and autumn periods and the Warring States period, “Yang moves and disperses, so it changes Qi, Yin is quiet and tranquil, so it takes shape.” Moxibustion can nourish Qi, warm yang, remove blood stasis, and dredge collaterals. Modern research shows that warm needling is better than single acupuncture [103], and thunder fire Moxibustion plus acupuncture is better than single acupuncture [104]. Clinical studies have shown that moxibustion has a certain role in promoting ovulation [105,106], thus regulating the menstrual cycle. Moxibustion can increase the thickness of endometrium, improve the level of serum sex hormone, and regulate the ovulation cycle in the treatment of PCOS with menstrual disorders. At the same time, it may be related with adjusting the levels of serum tumor necrosis TNF- *α* and nuclear transcription NF KB in patients with PCOS. A recent randomized controlled trial has shown that heat-sensitive moxibustion can significantly improve ovulation and correct menstrual disorders in women who have ovulation disorders. In this study, 70 patients were randomly divided into the control group and the observation group, with 35 patients in each group. On the basis of the control group, the observation group was added with heat-sensitive moxibustion, and the heat-sensitive moxibustion was carried out at Guanyuan, uterus, Xuehai, and Shenshu, every other day for 10 minutes. After 6 menstrual cycles, the levels of serum T and LH in the observation group were lower than those in the control group, while the E2 level was higher in the observation group than in the control group (*P* < 0.05). However,owing to the lack of the mechanism of moxibustion treating with menstrual disorders, we need more scientific basis researches to guide the clinical application.

### 3.3. Application of Acupoint Catgut Embedding in PCOS Patients with Menstrual Disorders

Acupoint catgut embedding (ACE) is based on the theory of acupuncture and moxibustion. It can achieve the purpose of stimulating the meridians and balancing Yin and Yang by placing absorbable sheep gut suture at acupoints with needle and medicinal thread. In the view of Western medicine, ACE therapy is considered as an exogenous invasive therapy [13,107], leading to the mechanism of its effectiveness and safety not being clarified; so, the clinical trials related to ACE have totally been completed in China. According to a recent randomized controlled trial, 84 PCOS obese patients with delayed menstruation and IR were selected by Gui-Zhi et al. [108] and divided into the observation group and control group, with 42 patients in each group. The observation group was additionally given acupoints such as BL20, BL23, ST25, GB34, ST40, ST36, SP6, etc., on the basis of the control group. After three months, the BMI of the observation group was lower than that of the control group, and the IR was significantly improved. Related clinical studies [82] reported that the corresponding body segments of acupuncture for ovarian domination were TH12-L2 and S2-S4 [109]. Therefore, ACE was mainly implanted in the muscles of abdomen and lower limbs [13], which inhibited the activity of sympathetic nervous system [110], thus promoting ovulation in PCOS patients, and improving hyperandrogenemia and menstrual disorder. Current animal experiments have shown that ACE may regulate menstruation in PCOS patients by reducing serum T level. The team of Tian et al. and Yi et al. studied the PCOS rats induced by TP and HCG under ACE treatment, and serum LH and T levels were significantly decreased after intervention [13, 111, 112]. Both IR and serum androgen level was decreased [112–114] by regulating the level of reproductive endocrine hormone. At the same time, some studies suggested that ACE may achieve therapeutic effect through the dual stimulation of suture in the process of decomposition and absorption [115]. As for the effective acupoints for catgut embedding, relevant studies [116–118] believed that the main acupoints (ST25, ST40, ST36, SP6, SP9, BL20, BL23, CV4, CV6, CV12) belong to the stomach meridian of Foot Yangming, spleen meridian of foot Taiyin, bladder meridian of foot Taiyang, and Ren Mai, which provide modern medical basis for the accuracy of acupoint selection. Although ACE therapy has the advantages of simple operation, it still lacks the theoretical basis of evidence-based medicine; in addition, the safety of its operation is still questionable. Consequently, higher quality and more rigorous experimental studies are needed to explore the potential mechanism.

For summarizing the randomized clinical trials, please refer to Table 2 and Figure 1.

## 4. Other Treatments for PCOS Menstrual Disorders

### 4.1. Auricular Points

Auricular points, also known as reaction points and stimulation points [81], are the acupoints distributed on the auricle. Local reactions often occur in certain parts of the auricle generally when the human viscera or body is in a state of illness, such as tenderness, nodules, discoloration, electrical conductivity, etc. This phenomenon can be used as a reference for the diagnosis of diseases, or to stimulate these reaction points (ear points) to prevent and treat diseases. The ear is closely related to viscera and meridians; there are corresponding reaction areas (auricular points) in the auricle of viscera. Thus, stimulating auricular points has an effect of partly regulating the corresponding viscera [119]. The main methods of stimulating auricular points are acupuncture, embedding needle, bloodletting, auricular point sticking, magnetic therapy, massage, etc.

The auricular points for treating PCOS with menstrual disorders are: the uterus, the ovary, the kidney, the liver, the spleen, and the pelvic cavity. Auricular acupoints are commonly used for auricular point sticking [120]. The main acupoints include liver, kidney, uterus, ovary, and endocrine, and the matching points are spleen, waist, and pelvic cavity. Appropriate acupoints are selected according to the TCM syndrome differentiation. After the acupoints are disinfected, the auricular plaster is applied to the sensitive parts of auricular points, pressing 3–4 times a day for 30 s each time. Use your fingers to knead, press, pinch, and press the sensitive parts of auricular points to the degree of strong acid, distension, heat, and pain. Change the auricular acupoint plasters every 3 days, 30 days as a course of treatment. Studies have shown that [121] 18 cases of 26 patients with irregular menstruation were cured after undergoing the treatment of accounting the main points of uterus, endocrine, internal genitalia, kidney, and liver, combined with the waist, pelvic cavity, and spleen, accounting for 69.2%. Based on auricular acupuncture and the principle of vagus nerve stimulation (VNS), a noninvasive transcutaneous vagus nerve stimulation (ta-VNS) was established. The results of comparative study on four VNS indications showed that ta-VNS has almost the same effect as VNS. In addition, ta-VNS has the advantages of security, economy, and portability. In the aspect of clinical prevention and treatment of PCOS, polycystic ovary (PCO) is the target organ, and the nerve and vagus nerve are related to it. In general, regulating the reproductive and endocrine disorders of PCOS from the perspective of vagus nerve, introducing at-VNS into the treatment of PCOS, and improving acupuncture therapy such as ear acupuncture may become the new research directions [122].

### 4.2. Massage

Massag is a method which is based on “the theory of viscera and meridians of TCM” and combined with “the anatomy and pathological diagnosis of Western medicine.” The operation method of massage is to select appropriate manipulation and then act on specific parts of the body surface to regulate the physiological and pathological conditions of the body achieving the purpose of physical therapy [123]. Massage has various functions of relaxing muscles and bones, reducing pressure, promoting the blood circulation, detoxification, etc. [124]. The scope of gynecological massage treatment includes menstrual diseases (irregular menstruation, dysmenorrhea), leucorrhea, postpartum diseases (breast carbuncle, etc.), gynecological miscellaneous diseases (hyperplasia of mammary glands, menopausal syndrome), etc. [125].

Modern women's life, work, family, and other aspects of pressure will make some of them suffer from PCOS, resulting in menstrual disorders which are physiological, not necessarily with drug treatment. Thus, PCOS patients with menstrual disorders using massage therapy is an excellent method. The massage methods for treating PCOS with menstrual disorders include: hand acupoints massage for menstrual disorders, foot acupoints massage for menstrual disorders, head acupoints massage for menstrual disorders, ear acupoints massage for menstrual disorders, and whole body acupoints massage for menstrual disorders. In general, whole body acupoint massage is often used to treat menstrual disorders. TCM pays attention to holistic concept and syndrome differentiation and treatment. For acupoint massage, the patients with liver qi stagnation are treated with tonifying the kidney (Guanyuan, Shenshu, Taixi), soothing the liver (Taichong, Guanyuan, Sanyinjiao), warming Yang (Guanyuan, Qihai, moxibustion Yinbai), and activating blood (Diji, Xuehai, Zhongji); those with deficiency of kidney qi are treated with tonifying the kidney (Guanyuan, Shenshu, Taixi), promoting qi (Guanyuan, Qihai, Zusanli, Sanyinjiao), and promoting blood circulation (Diji, Xuehai, Zhongji); Patients with phlegm obstruction are treated with tonifying the kidney (Guanyuan, Shenshu, Taixi), resolving phlegm (Fenglong, Guanyuan, Uterus, Sanyinjiao), and activating blood (Diji, Xuehai, Zhongji). Three consecutive treatments were administered, namely, 3rd day after menstruation, 12th to 15th day of menstrual cycle, 18th to 21st day, and 2nd to 5th day after menstruation. After 3–6 months of continuous treatment, menstrual disorders were significantly improved [126]. The study showed that [127] raising your legs and massaging Sanyinjiao point which is located three inches above from the tip of the medial malleolus of the foot (about four transverse fingers) for 49 times with your thumb meanwhile against the posterior edge of the tibia can help smoothen the flow of Qi and blood and strengthen the spleen at the same time benefiting Qi, which are contributed to improve the symptoms of menstrual disorders.

### 4.3. Qigong

Qigong is a traditional Chinese method of healthcare, health preservation, and disease elimination. In ancient times, the content of Qigong was very extensive. Qigong is characterized by the combination of mind, Qi, and body through the subjective efforts of practitioners. It mainly includes the adjustment of breathing, physical activities, and consciousness, including the means of regulating breath, body, and mind, with the purpose of strengthening the body, preventing and treating diseases, keeping fit and prolonging life, and developing the potential of physical and mental exercise. Heart regulation is to regulate psychological activities, breath regulation is to regulate respiratory movement, and body regulation is to regulate body posture and movement. These three regulations are the basic methods of Qigong exercise and are called the three elements or basic norms of Qigong discipline [128]. Qigong focuses on calmness, relaxation of body and mind, and the peace of mind, which are of great benefit to the harmony of Qi and blood in women, and the smooth flow of Qi also improves the self-regulation of blood. Due to the modern women's fast-paced life, the urgent need is to regulate the pace of life, and to calm down your body and mind. This has a considerable effect on improving PCOS menstrual disorder [129]. The aim of this study [130] is to observe the effect of “fitness Qigong six character formula” on menstrual disorders of female college students [130]. Eighty-six female college students with PCOS menstrual disorders were randomly selected. 46 cases were classified as Qigong group. After 12 weeks of Qigong exercise, the symptoms of menstrual disorders of 41 cases got different degrees of improvement. The total effective rate was 89.1%. Among the 18 students with symptoms of abnormal menstrual cycle, 16 cases were improved, and the effective rate was 89%; in 15 cases of abnormal menstrual volume, 14 cases were improved, and the effective rate was 93%; in 13 cases of abnormal menstrual period, 11 cases were improved, the effective rate was 84%. In the control group, 3 cases among 40 students in the control group improved their symptoms, and the effective rate was 7.5%. The other 37 cases had no obvious improvement. It can be concluded that: Fitness Qigong six character formula can effectively improve the symptoms of irregular menstruation of PCOS female college students.

### 4.4. Tai Chi

The core of Taiji theory is yin-yang theory. The yin-yang fish in Taiji diagram symbolizes the state of things moving in cycles. The basal body temperature curve of female normal menstrual cycle showed periodic biphasic changes. Similarly, Taiji diagram is often used to show the physiological and pathological changes of menstruation [131]. According to the description of menstrual mechanism in Neijing, the maintenance of menstruation is affected by age, viscera, Tiangui, and other parameters. Besides, the whole set of Taijiquan moves continuously in a gentle and soothing way, like silkworms spinning in spring. And the continuous movement makes the whole body's meridians unblocked, resulting in the smooth circulation of Qi and blood thereby improving the microcirculation of the human body and physique [132]. The characteristics of Taiji are as follows: (1) The combination of will and form, focusing on will. (2) The combination of mobile and interval focuses more on interval. (3) Moving along meridians and exaggerate arm rotation. (4) Selecting the acupoints along the meridians, with fingers instead of needles. (5) In the whole process, removing the disturbance of all the evil thoughts so as to achieve the state of meditation and stabilize your mind, meanwhile, keeping your body highly relaxed [133]. Consequently, Tai Chi is outstanding in regulating Yin and Yang and regulating qi for women. Women are innate with blood. Only when the Yin and Yang are balanced, Qi and blood are smooth, can menstruation naturally occur on schedule; thereby, Tai Chi plays a great role in regulating the menstruation of women, and has a significant effect on PCOS menstrual disorders. Li. et al.'s research showed [133] that Taijiquan involved training for 60 minutes each time, three times a week for 12 weeks, based on the women's initial physical activity level. Each session includes 10 minutes of warm-up and cooling. It adopts 24 kinds of Simplified Taijiquan recommended by the General Administration of sport of China. Finally, the weight, waist/hip circumference, and blood pressure of PCOS patients with menstrual disorders before and after Taijiquan treatment were significantly improved, and the insulin level was significantly improved, which will eventually promote the improvement of menstruation.

### 4.5. Cognitive Behavioral Therapy

Changes in hormone levels in PCOS and Negative Affectivity (NA) are intricately linked. Relevant experiments showed [134] that LH is significantly negatively correlated with Hospital Anxiety and Depression Scale (HADS), and FSH is significantly negatively correlated with Hospital Anxiety and Depression Scale-Anxiety (HADS-A); a slight increase in free T can reflects a higher degree of depression. Therefore, due to the influence of hormone level, the economic burden of diseases treatment and the potential social doubt, PCOS patients will bear more self-doubt and external disagreement, which may lead to more tension, anxiety, and even depression.

In view of the origin of anxiety, depression, and bad emotions of PCOS patients, Leah Brennan's research team [135] proposed the following psychotherapy strategies: clinicians consciously assist patients to learn and establish the following modes: goal setting, self-monitoring, cognitive reconstruction, problem-solving, and recurrence prevention. In the process of adjuvant treatment, through motivational interview, self-monitoring, and time management strategies, we should teach patients behavior changing skills and coping skills of life and work, so as to improve their ability to deal with bad emotions and restore their mental health; at the same time, we should seek help from psychologists and other professionals as appropriate.

Mindfulness-based therapy, rooted in Buddhist meditation [136], is one of the ways of psychological intervention. By strengthening the relaxation response of the human body, it may effectively prevent common mental diseases (including depression and anxiety), relieve the stress and anxiety in life and work, and assist people to maintain a healthy mental state [137]. In Norio Watanabe's research team [138], experimental groups were given mindfulness pressure management plan for 8 weeks. The final experimental results were satisfactory; the scores of HADS, perceived stress scale, and quality of daily life questionnaire (Questionnaire on the degree of perplexity of patients with PCOS clinical characteristics, such as hirsutism, obesity, menstrual disorders, etc.) in the experimental group were significantly decreased; and the scores of HADS (Dass 21) were significantly decreased. Dass 21 assesses the state of the sympathetic nervous system rather than the subjective pressure of the patient, which further illustrates the influence of mindfulness therapy on PCOS patients.

Behavioral cognitive therapy is safe and has no side effects, which can effectively relieve the negative emotions of depression and anxiety in patients with PCOS, assist patients to adjust to the overall state of the body from the psychological level, and help shorten the treatment cycle of the disease.

### 4.6. Establishment of Healthy Life Mode

According to International evidence-based guideline for the assessment and management of PCOS, weight control is one of the main treatment strategies for PCOS [139]. A healthy lifestyle for women with PCOS includes taking a low carbohydrate and ketogenic diet; taking ancient diet or less processed food, cereal, or dairy products; hypoglycemia diet; regular physical exercise; adequate sleep; practicing decompression technology; and various forms of online and written learning courses [140]. However, it should be noted that weight loss through short-term diet and exercise is only the beginning of cultivating a healthy lifestyle. It is the management of long-term lifestyle and the maintenance of healthy eating habits that cannot be ignored. The Mediterranean Diet (MD) is an anti-inflammatory diet, including regular consumption of unsaturated fat, low glycemic index carbohydrates, fiber, vitamins and antioxidants, and an appropriate diet [141]. Luigi Barrea's team [142] found that MD had positive effects on T level and Ferriman-Gallwey score of PCOS patients to varying degrees; and the intake of complex carbohydrates, fiber, monounsaturated fatty acids, and n-3 polyunsaturated fatty acids was positively correlated with C-reactive protein level; compared with the control group, patients with PCOS on a MD had a lower intake of these substances. MD can reduce the inflammatory state, help patients with PCOS-IR and hyperandrogenemia to reduce the inflammatory state of the body. Therefore, it is recommended that the MD be used for PCOS patients.

Apart from reducing exogenous intake, an increase in the amount of exercise is required for weight loss. Relevant experiments have proved that exercise has a certain effect on the recovery of favorable metabolic and ovarian functions [9,143]. The study [144] found that after 12 weeks of exercise, the BMI and Cardiovascular Health Index of the exercise group were improved, and the levels of the Anti-Müllerian Hormone of Ovary (AMH) and Malondialdehyde (MDA) were decreased, while the levels of Superoxide Dismutase (SOD) and Total Antioxidant Capacity (TAC) were increased. Some researchers [145] found that it is the intensity of exercise rather than the amount of the exercise is the key to the patients who want to improve their state by exercising. In addition, the researchers found that patients with PCOS should have at least 120 minutes of vigorous exercise every week for at least 10–12 weeks to better improve the status of PCOS.

### 4.7. Complementary Alternative Medicine

In recent years, the research on complementary and alternative drugs has made some progress in the treatment of PCOS, such as probiotics, melatonin, fish oil, fatty acid, vitamin D, vitamin K, carnitine, chromium, selenium. They are found to have certain intervention effects on PCOS [146].

The microbial regulation of Lactobacillus may be an effective way to alleviate PCOS. The results showed that Lactobacillus could alleviate the symptoms of PCOS in letrozole model rats [147]. Some *Lactobacillus* may play an important role in improving PCOS by regulating and controlling the sex hormone-related microorganisms. Melatonin [148] has certain effects on reducing oxidative stress, and acts as an antioxidant, anti-inflammatory, and antidepressant agent; fish oil [149] can significantly improve the expression of PPAR - *γ*, IL-1, and IL-8 genes related to PCOS; omega-3 fatty acids [149] can reduce C-reactive protein and increase adiponectin levels to reduce the inflammatory state of women with PCOS; after providing PCOS patients with vitamin D deficiency supplements including calcium, vitamin D, and K for 8 weeks, insulin metabolism, serum triglyceride, and very low-density lipoprotein cholesterol levels were improved [150]; combining the use of carnitine with chromium [151] can improve the mental health indicators, serum total T, high-sensitivity C-reactive protein, Total Antioxidant Capacity (TAC), and Malondialdehyde (MDA) levels of women with PCOS, as well as the gene expression of Interleukin-6 (IL-6) and Tumor Necrosis Factor alpha (TNF - *α*); selenium (SE) [152] is a kind of antioxidant and has an insulin-like effect. All of the drugs listed above may have a therapeutic effect on PCOS and provide new direction for the treatment of PCOS.

## 5. Summary

Owing to the wide range of applications, CAM receives more and more attention and application at home and abroad, and PCOS menstrual disorders are also paid more and more attention by CAM. CAM is a kind of therapy which has less erosion and fewer side effects on patients, providing more beneficial choices for patients and opening up a broad path to health [153]. For PCOS menstrual disorders, TCM and acupuncture are the most commonly used CAM methods, but other CAM medicine therapies also help improve PCOS menstrual disorders [154]. However, at present, CAM is still in the early stage of the trial, which means that there are still some limitations. We should also collect a large number of samples for large-scale clinical research, especially for cognitive behavioral therapy [137], healthy life model [139], replacement drugs [146], etc., and we should further explore its therapeutic potential in the future research [155].

## Figures and Tables

**Table 1:** The summary of randomized studies of Chinese medicinal treatment on PCOS menstrual disorders.

Study ID	Design	Sample size (human)	Interventions	Treatment duration	Outcomes	Limitation
26	RCT	100	Treatment arm: the tanshinone capsules Control arm: no intervention	12 weeks	Treatment arm: not mentioned Control arm: not mentioned	Small sample size
33	Controlled trial	100	Treatment arm: BBR Control arm: no intervention	6 months	Treatment arm: effective rate, 48.3 % (22 of 50) Control arm: no changes	Not mentioned blindness Not mentioned placebo
35	RCT	150	Treatment arm: BBR Control arm: metformin Placebo: no intervention	3 months	Treatment arm: effective rate, 48.6 % (18 of 37) Control arm: effective rate, 36.8 % (14 of 38) Placebo: effective rate, 20.6 % (7 of 37)	Small sample size
40	RCT	15	Treatment arm: cinnamon Placebo: no intervention	3–6 months	Treatment arm: not mentioned Control arm: not mentioned	Small sample size
41	RCT	66	Treatment arm: cinnamon Placebo: no intervention	12 weeks	Treatment arm: not mentioned Placebo: not mentioned	Small sample size
42	RCT	15	Treatment arm: cinnamon Placebo: no intervention	8 weeks	Treatment arm: not mentioned Placebo: not mentioned	Small sample size
46		58	Treatment arm: Cangfu Daotan decoction Control arm: metformin	6 months	Treatment arm: effective rate, 82.7% (24 of 29) Control arm: effective rate, 65.5% (19 of 29)	Not mentioned blindness Not mentioned placebo
47		68	Treatment arm: clomiphene and Cangfu Daotan decoction Control arm: clomiphene	6 months	Treatment arm: effective rate, 88.6 % (31 of 35) Control arm: effective rate, 84.8 % (28 of 33)	Not mentioned blindness Not mentioned placebo
49		60	Treatment arm: Cangfu daotan decoction and Diane-35 Control arm: no intervention	3 months	Treatment arm: effective rate, 81.6 % (49 of 60) Control arm: not mentioned	Not mentioned blindness Not mentioned placebo Not mentioned control group
52		66	Treatment arm: Zigui decoction Control arm: Diane-35	3 months	Treatment arm: effective rate, 45.45 % (15 of 33) Control arm: effective rate, 21.21 % (7 of 33)	Not mentioned blindness Not mentioned placebo
53		40	Treatment arm: standardized Chinese herbal medicine Control arm: no intervention	6 months	Treatment arm: effective rate, 52.6 % (10 of 19) Control arm: not mentioned	Not mentioned blindness Not mentioned placebo Not mentioned control group
54		60	Treatment arm: metformin, Dian-35 and danzhi xiaoyao pill Control arm: metformin and Dian-35	3 months	Treatment arm: effective rate, 87.5 % (21 of 24) Control arm: effective rate, 58.3 % (14 of 24)	Not mentioned blindness Not mentioned placebo
55		48	Treatment arm: Longdan xiegan decoction Control arm: Diane-35	3 months	Treatment arm: effective rate, 82.6 % (19 of 23) Control arm: effective rate, 78.26 % (18 of 23)	Not mentioned blindness Not mentioned placebo
56		22	Treatment arm: Tiangui decoction Control arm: metformin	3 months	Treatment arm: effective rate, 60 % (6 of 10) Control arm: effective rate, 33.3 % (4 of 12)	Not mentioned blindness Not mentioned placebo

**Table 2:** The summary of randomized studies of acupuncture treatment on PCOS menstrual disorders.

Study ID	Design	Sample size (human)	Interventions	Treatment duration	Outcomes	Limitation
72	RCT	86	Treatment arm: Abdominal acupuncture Control arm: metformin	6 months	1. HOMA-IR were reduced significantly in the two groups (*P* < 0.05).2. Menstrual frequency, HDL-C, follicle-stimulating hormone increased in both groups (*P* < 0.05).	Lack of sham methods
74	RCT	342	Treatment arm: true acupuncture + metformin placebo Control arm: sham acupuncture + metformin, sham acupuncture + metformin placebo	4 months	Treatment arm: HOMA-IR decrease 25% Control arm: HOMA-IR decrease 5%	The limitation of being a single center study without comparison groups
76	RCT	181	Treatment arm: acupuncture group Control arm: same physical therapists	10–13 weeks	Treatment arm: effective rate, 62.5% (10 of 16) Control arm: effective rate, 41.6% (5 of 12)	
77	RCT	60	Treatment arm: Tung's acupuncture group Control arm: CPA/EE group	12 weeks	Treatment arm: LH/FSH ratio −0.66, *P* < 0.001 Control arm: not mentioned	Clinical improvement of symptoms was not examined in this study
78	Nonrandomized, longitudinal, prospective study	60	Treatment arm: electro-acupuncture group Control arm: No intervention	3 months	Treatment arm: effective rate, 37.5 % (9 of 24) Control arm: not mentioned	The sample size is small
80	RCT	84	Treatment arm: low-frequency electro-acupuncture Control arm: no intervention	16 months	Treatment arm: effective rate, 72.9% (43 of 59) Control arm: effective rate, 33.3% (5 of 15)	Low success rate to confirm ovulation (data not shown)
81	RCT	84	Treatment arm: electro-acupuncture group; Physical exercise Control arm: no intervention	32 weeks	Treatment arm: effective rate, 74.4% (32 of 43) Control arm: effective rate, 48.7% (20 of 41)	The sample size is small
82	RCT	32	Treatment arm: acupuncture group Control arm: attention control	4 months	Treatment arm: effective rate, 68.75 % (11 of 16) Control arm: effective rate, 37.5 % (6 of 16)	
88	RCT	143	Treatment arm: true acupuncture Control arm: sham acupuncture	5 months	Treatment arm: effective rate, 57.5% (23 of 40) Control arm: effective rate, 47.7 % (21 of 44)	
109	RCT	70	Treatment arm: heat-sensitive moxibustion plus clomifene citrate capsules Control arm: oral clomifene citrate capsules	6 months	Treatment arm: effective rate, 51.5% (18 of 35) Control arm: effective rate, 20 % (7 of 35)	The sample size is small
111	RCT	84	Treatment arm: metformin and acupoint thread-embedding Control arm: oral metformin	3 months	Treatment arm: effective rate, 87.5 % (35 of 40) Control arm: effective rate, 65.9 % (27 of 41)	The sample size is small
